# Meta-analysis of retinal transcriptome profiling studies in animal models of myopia

**DOI:** 10.3389/fmed.2024.1479891

**Published:** 2025-01-14

**Authors:** Teele Palumaa, Shruti Balamurugan, Machelle T. Pardue

**Affiliations:** ^1^Department of Ophthalmology, Emory University, Atlanta, GA, United States; ^2^Institute of Genomics, University of Tartu, Tartu, Estonia; ^3^Atlanta VA Center for Visual and Neurocognitive Rehabilitation, Decatur, GA, United States; ^4^Department of Biomedical Engineering, Georgia Institute of Technology, Atlanta, GA, United States

**Keywords:** myopia, transcriptomics, meta-analysis, experimental models, RNA-Seq

## Abstract

**Objective:**

Myopia prevalence is increasing at alarming rates, yet the underlying mechanistic causes are not understood. Several studies have employed experimental animal models of myopia and transcriptome profiling to identify genes and pathways contributing to myopia. In this study, we determined the retinal transcriptome changes in response to form deprivation in mouse retinas. We then conducted a transcriptome meta-analysis incorporating all publicly available datasets and analyzed how the results related to the genes associated with refractive errors in human genome-wide association studies (GWAS).

**Methods:**

Form deprivation was induced in three male C57BL6/J mice from postnatal day 28 (P28) to P42. Retinal gene expression was analyzed with RNA sequencing, followed by differential gene expression analysis with DESeq2 and identification of associated pathways with the Kyoto Encyclopedia of Genes and Genomes (KEGG). A systematic search identified four similar retinal transcriptomics datasets in response to experimental myopia using chicks or mice. The five studies underwent transcriptome meta-analyses to determine retinal gene expression changes and associated pathways. The results were compared with genes associated with human myopia.

**Results:**

Differential gene expression analysis of form-deprived mouse retinas revealed 235 significantly altered transcripts, implicating the BMP2 signaling pathway and circadian rhythms, among others. Transcriptome-wide meta-analyses of experimental myopia datasets found 427 differentially expressed genes in the mouse model and 1,110 in the chick model, with limited gene overlap between species. Pathway analysis of these two gene sets implicated TGF-beta signaling and circadian rhythm pathways in both mouse and chick retinas. Some pathways associated only with mouse retinal changes included dopamine signaling and HIF-1 signaling pathway, whereas glucagon signaling was only associated with gene changes in chick retinas. The follistatin gene changed in both mouse and chick retinas and has also been implicated in human myopia. TGF-beta signaling pathway and circadian entrainment processes were associated with myopia in mice, chicks, and humans.

**Conclusion:**

This study highlights the power of combining datasets to enhance statistical power and identify robust gene expression changes across different experimental animal models and conditions. The data supports other experimental evidence that TGF-beta signaling pathway and circadian rhythms are involved in myopic eye growth.

## 1 Introduction

Myopia prevalence rates are increasing worldwide (1) yet the causes for this are not fully known. While there is convincing evidence that the retina is essential for signaling refractive eye growth [see review (2)], the exact mechanisms that regulate this process remain elusive. Current myopia control methods, which include optical and pharmacological treatments and lifestyle changes, are limited in their effect size (3). Therefore, understanding the causal mechanisms regulating refractive development and myopia is essential for optimizing current treatments and developing novel approaches to myopia management.

The field of myopia research has significantly benefitted from studies conducted on animal models that investigate molecular changes associated with myopia development. Two primary methods for inducing experimental myopia are form deprivation and lens-induced myopia, where either a diffuser goggle or a negative-powered lens is placed in front of the experimental eye, resulting in the induction of myopic refractive shift (4, 5). The increasing accessibility and affordability of advanced sequencing technologies have made transcriptome analyses increasingly popular. Several studies have implemented transcriptome profiling of the retinal tissue to identify molecular signatures and cellular pathways associated with myopic eye growth (6–13). However, despite the wealth of data generated, the sample size of each study is usually relatively small, which can limit the statistical power and robustness of findings. In addition to the variability between studies arising from slight differences in experimental protocols, other sources of variation include differences in sequencing methods and analysis pipelines (14). Furthermore, the robustness of retinal changes in response to myopic stimulus can also be evaluated by analyzing samples from different species.

Conducting meta-analyses addresses some of the limitations of transcriptome profiling by retrieving samples from multiple studies that analyze the same experimental condition and applying the same analysis pipelines. Meta-analyses remove variation introduced by applying different analysis pipelines and increase the sample size, thus enhancing statistical power. This approach provides an overview of the most robustly changing genes and pathways across different laboratories and experimental models and has been used in several contexts (15, 16). Identifying these consistent changes can result in a better understanding of the underlying biological processes and mechanisms.

In this article, we first present a transcriptome analysis of retinal changes in a mouse model of form deprivation myopia (FDM). Then, we perform a systematic search and meta-analysis of publicly available transcriptomic datasets related to experimental myopia in animal models. Through this combined approach, we aim to identify key gene expression changes and pathways involved in myopia development, which may provide insights into the robust molecular mechanisms underlying this condition. Furthermore, we compare the transcriptomic changes with genes and pathways associated with human refractive errors to determine which retinal changes in experimental myopia may be associated with the human disease.

## 2 Materials and methods

### 2.1 Animals

All animal experiments were approved by the Atlanta Veterans Affairs Institutional Animal Care and Use Committee (protocol V015-14). Male C57BL/6J mice were housed in a standard 12-12 hour light-dark cycle. Food and water were provided *ad libitum*. From postnatal day (P) 28, mice were exposed to FDM by surgically attaching a head-mounted diffuser goggle over the right eye (17). The left eye remained uncovered and served as an internal control. Refractive error, corneal curvature and axial length were measured at P28, P35, and P42. After dilating the eyes with 1% tropicamide, mice were anesthetized (ketamine 80 mg/kg; xylazine 16 mg/kg). Refractive error was measured with an automated infrared eccentric photorefractor (5), corneal radius of curvature was measured using an automated keratometer (18) and axial length with a spectral domain optical coherence tomography system (Bioptigen, Durham, NC, USA). Myopic shift was calculated as the refractive error difference between the goggled and uncovered eye.

### 2.2 Tissue preparation and RNA sequencing

At P42, mice were sacrificed by cervical dislocation during the light phase; their retinal tissue was immediately collected, flash-frozen on dry ice, and stored at −80°C until further processing. Total RNA was extracted using TRIzol and RNeasy Micro kit RNA extraction methods according to the manufacturers' protocols. RNA quantity was measured with a NanoDrop 1000 (Thermo Fisher Scientific), RNA quality was analyzed with the Agilent 2100 Bioanalyzer using the Pico chip (Agilent Technologies), and samples with an RNA integrity number (RIN) >7.5 were used for RNA sequencing (RNA-Seq). RNA was submitted to Emory Integrated Genomics Core. Following poly-A enrichment, 50-base paired-end libraries were prepared and sequenced on the Illumina HiSeq Sequencing System at 50 M reads per sample. The RNA-Seq data of this study are available in the Gene Expression Omnibus repository, accession number GSE284642, and BioProject, accession number PRJNA1200000.

### 2.3 RNA sequencing data analysis

FASTQ files were uploaded to the Galaxy web platform (19). Read quality was analyzed with FastQC (20). Reads filtered for low-quality reads and trimmed using Trimmomatic (21). Transcript abundance was quantified with Salmon (22). Count normalization and differential analysis were conducted with DESeq2 (23) in R (24) and an unadjusted *p* value of < 0.05 was considered statistically significant, as this analysis is exploratory in nature with an aim to suggest further hypotheses. Differentially regulated pathways and cellular functions were further analyzed using the KEGG database (25) and GO terms (26) and visualized with pathfindR (27) and ggplot2 (28).

### 2.4 Systematic search of transcriptomics datasets

A search was conducted on the PubMed, Gene Expression Omnibus (GEO) and BioProject databases using the following search statement: retina^*^ AND (transcriptom^*^ OR “RNA seq^*^” OR “RNA-seq^*^” OR microarray) AND (myopia OR “short^*^sighted^*^” OR “refractive error” OR “refractive development” OR “ocular growth” OR “eye growth” OR “experimental myopia” OR “lens-induced myopia” OR “image defocus” OR “form^*^deprivation”). All searches were conducted on June 5, 2024. The results were screened for studies that met the following inclusion criteria:

1. The study used an animal model.
2. The study included samples from control eyes and eyes with experimentally induced myopia.
3. The study analyzed retinal samples. All combined tissue samples, e.g. retina/RPE/choroid were excluded.
4. The study conducted whole-genome transcriptome profiling (RNA-Seq or microarray analysis).

### 2.5 Data processing and meta-analysis

With this search, six RNA-Seq datasets were identified (Table 1). The unprocessed data files of identified studies in the FASTQ format were located. In two instances, the studies filled the inclusion criteria, yet the raw data was not found nor accessed after contacting the corresponding author. Identified studies used either the chick or mouse model of myopia, and the samples from different species were analyzed separately.

**Table 1:** Experimental myopia retinal transcriptome profiling studies identified.

**#**	**References**	**BioProject accession number**	**Species**	**Myopia model**	**Animal age at the start of treatment**	**Myopia induction duration**	**Control group**	**No. of samples (experimental + control)**	**Sex**
1	Karouta et al. (7)	PRJNA678523	Chick	FDM	7 days	4 and 24 h	Age-matched untreated controls	15 (7 + 8)	M
2	Li et al. (2)	PRJNA832969	Mouse (C57BL/6J)	FDM	3 weeks	4 weeks	Contralateral untreated eye	12 (6 + 6, each sample 3 retinas pooled)	M
3	Shan et al. (45)	PRJNA766764	Chick	LIM (−10D)	4 days	24 and 48 h	Contralateral untreated eye	12 (6 + 6)	*Not collected*
4	Stone et al. (10)	PRJNA946718	Chick	FDM	Newly hatched	24–44 h (every 4 h)	Contralateral untreated eye	72 (36 + 36)	M and F
5	*Current article*		Mouse (C57BL/6J)	FDM	4 weeks	2 weeks	Contralateral untreated eye	5 (2 + 3)	M
**Not included**									
	Tkatchenko et al. (6)	–	Marmoset	LIM (−5D)	74 ± 5 days	10 days and 5 weeks	Contralateral eye with plano lens	24 (12 + 12)	M and F
	Ji et al. (9)	PRJNA994038 (*not found on June 5, 2024*)	Mouse (C57BL/6J)	LIM (−25D)	4 weeks	4 weeks	Contralateral untreated eye	6 (3 + 3)	*Not stated*

FDM, form-deprivation myopia; LIM, lens-induced myopia.

The FASTQ files were imported to the Galaxy platform (19), filtered for low-quality reads, and trimmed using Trimmomatic (21), specifying the adapter parameters to what was used in each experiment. The quality of individual reads was analyzed with FastQC (20) before and after the trimming process. Samples were excluded if the percentage of duplicates, read count, or GC content was >2 standard deviations from the mean of the respective study. Transcript abundance was quantified with Salmon (22), estimates were aggregated to the gene level.

Non-normalized untransformed count matrices of each study were obtained using tximport package (v 1.28.0) (29) in R. Next, ComBat-seq (30) from the sva package (v 3.48.0) (31) was used to remove batch effects between studies, specifying the study identifier as the batch variable and the treatment group (experimental myopia vs. control) as the grouping variable. Manipulations used for myopia induction were not separated in this meta-analysis. Low-abundance transcripts were eliminated by retaining only genes with >10 counts in the number of samples of the smallest experimental group. Differential gene expression between control and experimental myopia samples was conducted using the DESeq2 package (23). An unadjusted *p* value of < 0.05 was considered statistically significant, which does increase type I error risk, but as the analysis has inherently high biological variability, a more stringent criteria could eliminate potentially interesting and relevant findings. Transcripts were annotated using the *Mus musculus GRCm38* Ensembl v91 release or the *Gallus gallus* GRCg7b Ensembl v112 release genome assemblies. Differentially expressed pathways and cellular functions were further analyzed using the KEGG database (25), and visualized with pathfindR (27) and ggplot2 (28). Principal component analyses (PCA) of experiments were performed on the variance-stabilized counts of the RNA-Seq data using DESeq2 (23). PCA coordinates were extracted, considering treatment and batch as grouping factors.

Genes associated with refractive errors from genome-wide association studies (GWAS) were retrieved from the GWAS Catalog (32). Single nucleotide variants (SNVs) and their corresponding mapped genes were obtained for traits “myopia” (Experimental Factor Ontology [EFO] trait HP_0000545) and “abnormality of refraction” (EFO trait HP_0000539). Pathways enriched for genes associated with a genetic predisposition to myopia were determined using the KEGG database (25) and visualized with pathfindR (27) and ggplot2 (28).

## 3 Results

### 3.1 Transcriptomic changes in response to FDM in the mouse retina

**Figure 1:**
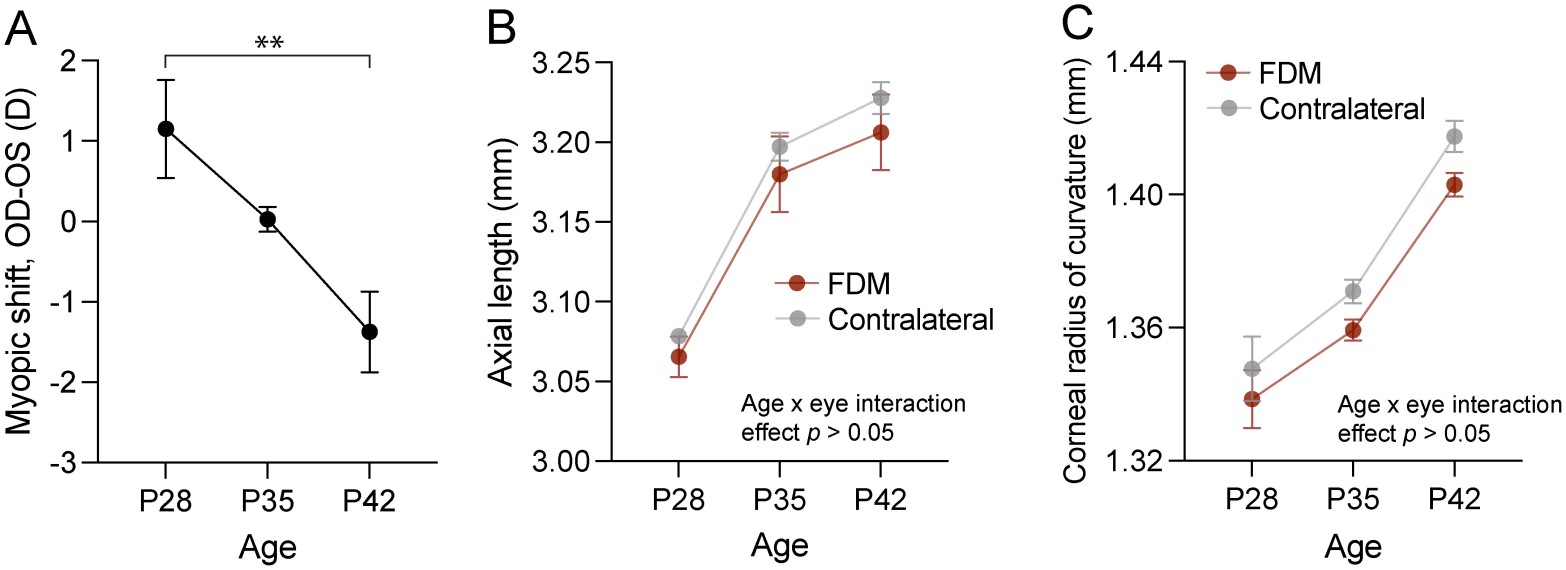
Ocular changes in response to form deprivation in mice used in the RNA sequencing experiment. **(A)** The myopic shift, expressed as the interocular difference in refractive error, showed relative myopia in the form-deprived eye of wild-type C57BL/6J mice after two weeks of form deprivation. Axial length **(B)** and corneal curvature **(C)** dynamics in response to form deprivation revealed no interaction effect between age and form deprivation. Data are mean ± SEM, *n* = 3. In **(A)**, statistical analysis was performed using one-way ANOVA with Šidák's correction for multiple comparisons. In **(B, C)**, two-way repeated measures ANOVA was used. ** *p* < 0.01. FDM, form-deprivation myopia.

Form deprivation was induced in three male mice from postnatal day (P) 28 by placing a translucent goggle in front of the right eye with a head-mounted pedestal, while the contralateral left eye remained uncovered and served as a control eye (17). By P42, the form-deprived eyes developed myopia, as their refractive error was on average 1.37 ± 0.87 D [mean ± standard deviation (SD)] more myopic than the contralateral uncovered eyes (P28 vs. P42 *p* = 0.008, one-way ANOVA with Šidák's correction for multiple comparisons; Figure 1A). We did not observe statistically significant changes in axial length and corneal radius of curvature between the control and form-deprived eyes (two-way repeated measures ANOVA, age x eye interaction effect *p* > 0.05; Figures 1B, C), which is not uncommon as the magnitude of changes is very small (33, 34).

**Figure 2:**
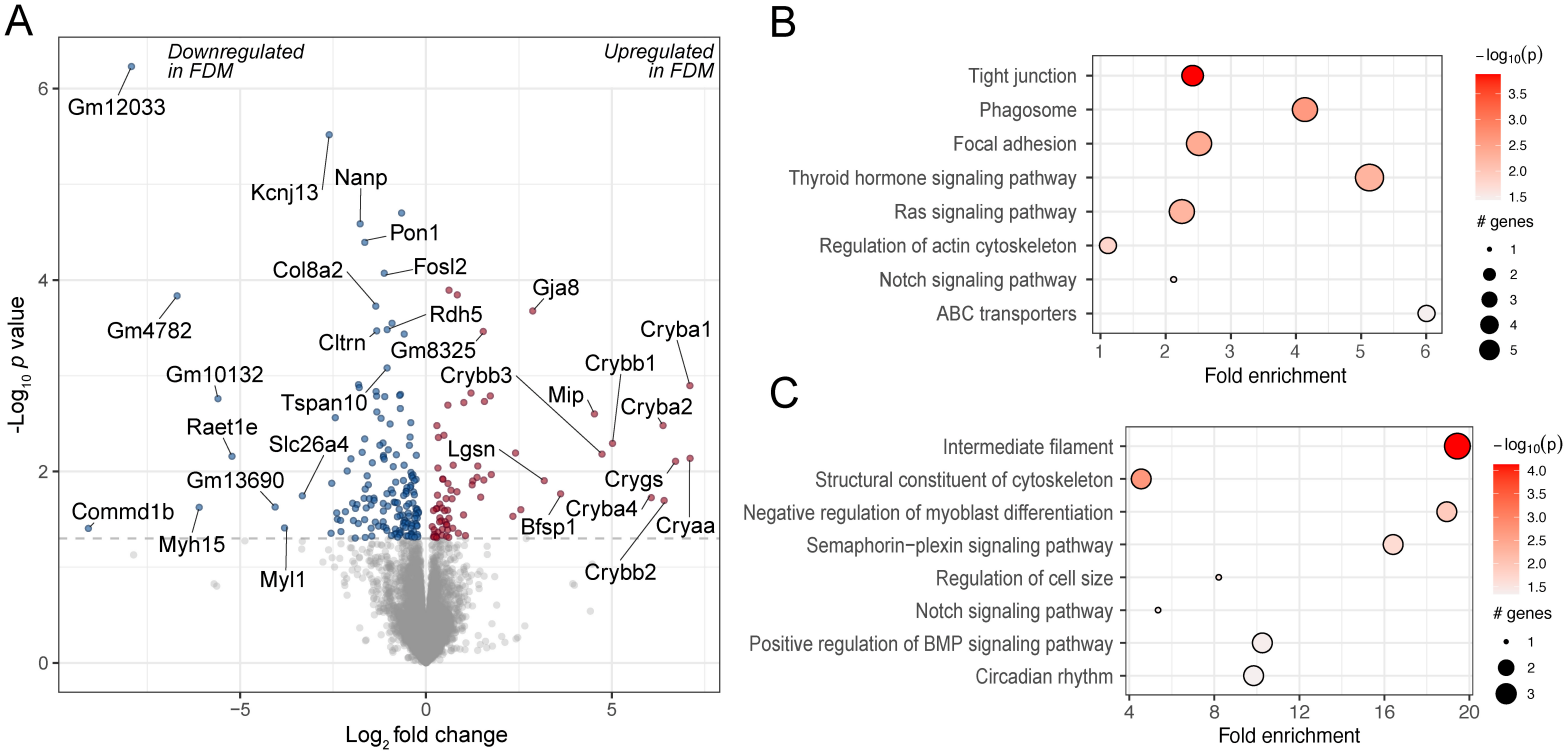
Differentially expressed genes and enriched pathways in FDM mouse retinas. **(A)** A volcano plot of genes differentially regulated in FDM retinas from wild-type mice, illustrating the log_2_ effect size and unadjusted log-transformed *p* values. The gray dashed horizontal line indicates an unadjusted *p* value of 0.05. Genes with significant changes are highlighted in red (upregulated) and blue (downregulated), with a selection of gene names indicated. A selection of KEGG pathways **(B)** and GO terms **(C)** enriched for the differentially regulated genes are highlighted.

The retinas of these animals were submitted for RNA-Seq analysis. Upon quality control, one sample was removed as its read duplication level was >2 SD from the mean of the study. Differential gene expression analysis showed that 235 transcripts were differentially expressed between the control and experimental retinas (Figure 2A and Supplementary Table 1). Among the most highly upregulated transcripts were several crystallins (*Cry*), such as *Cryaa, Cryba1, Cryba2, Cryba4, Crybb1, Crybb2* and *Crygs*. Upregulation of different crystallin transcripts has been also demonstrated previously in chick compound retina/RPE/choroid tissue in response to FDM (35) and in chick retinas after FDM and LIM induction (36). Among the downregulated genes were paraoxonase 1 (*Pon1*), an antioxidative protein associated with lower activity in AMD (37) and potentially linked to oxidative stress, which has been reported in myopia (38); and retinoic acid early transcript 1E (*Raet1e*), which is intriguing as retinoic acid signaling has been implicated in myopia (39).

The genes were further analyzed for enriched pathways using the KEGG database and GO biological process terms. The enriched pathways included various cell signaling pathways, e.g. thyroid hormone and Ras signaling pathways. Thyroid hormone signaling has also been implicated in prior studies investigating the retinal response to experimental myopia (10, 40). A key gene in the Ras signaling pathway, *RASGRF1*, has been associated with myopia (41), and there is evidence that its downstream pathway, the MAPK pathway, is differentially regulated in myopic retinas (11). They also included the regulation of the BMP signaling pathway, which has been associated with experimental myopia in previous studies in the retina and choroid (42), as well as the sclera (43, 44) (Figures 2B, C, Supplementary Tables 2, 3).

### 3.2 Identification of publicly available retinal transcriptomics datasets of experimental myopia

To increase the number of experimental samples and thereby increase the statistical power of our analysis, while simultaneously reducing the variation induced by conducting experiments in different laboratories in different species and using slightly different RNA-Seq protocols, we sought to combine and analyze all similar experiments conducted to date. We performed systematic searches in PubMed, GEO, and BioProject to identify studies employing transcriptome profiling to study retinal gene expression in response to experimental myopia in animal models. These searches yielded 71, 42, and 8 results, respectively. After screening the articles for inclusion criteria (see Materials and methods), we determined six suitable studies: two using mice (8, 9), three using chicks (7, 10, 45), and one using marmosets (6) as experimental animals. Four of the studies were included in the meta-analysis, as well as the RNA-Seq experiment presented earlier (Table 1).

There were several global differences between the studies conducted in mouse and chick models regarding the developmental stage of animals and the duration of myopia induction. The experiments conducted in mice used juvenile animals aged between 3 and 4 weeks, while chicks were as young as newly hatched to 7 days old. In mice, myopia was induced for a total of 2 to 4 weeks, while in chicks, the induction was limited to 4–48 h. Four of the five studies induced myopia with form deprivation, and one study in chicks used a −10D lens. Four studies used the contralateral eye as a control eye, while one used age-matched untreated animals. Three of the studies used only male animals, one used male and female, and the sex of animals in one study was not collected (see an overview in Table 1).

Since the chick and mouse samples differed not only in the experimental animal used but also in the developmental stage and myopia induction length, we conducted two separate meta-analyses, one including samples from mouse studies and one including all samples from chick studies. In these two sets of samples, we analyzed the effect of experimental myopia on the retinal transcriptome. A total of 9 control and 8 experimental myopia samples were identified using the mouse model, and 49 control and 50 experimental myopia samples in the chick model. After removing samples with outliers regarding sequencing read quality metrics (see Materials and methods; Supplementary Tables 4, 5), a total of nine control and seven myopia mouse samples, and 46 control and 45 myopia chick samples were included in the downstream analysis.

### 3.3 Meta-analysis of retinal transcriptomics datasets of experimental myopia

Samples from all included studies underwent the same analysis pipeline to ensure the results were directly comparable. Principal component analyses (PCA) of the individual studies did not indicate a clear clustering of the experimental samples by myopia treatment (Supplementary Figure 1). In studies that used the contralateral eye as a control, the samples from the same experimental animal were clustered together (Supplementary Figures 1B, C, E). In the only study using experimental animals of both sexes, there was clear clustering based on sex (Supplementary Figure 1D).

**Figure 3:**
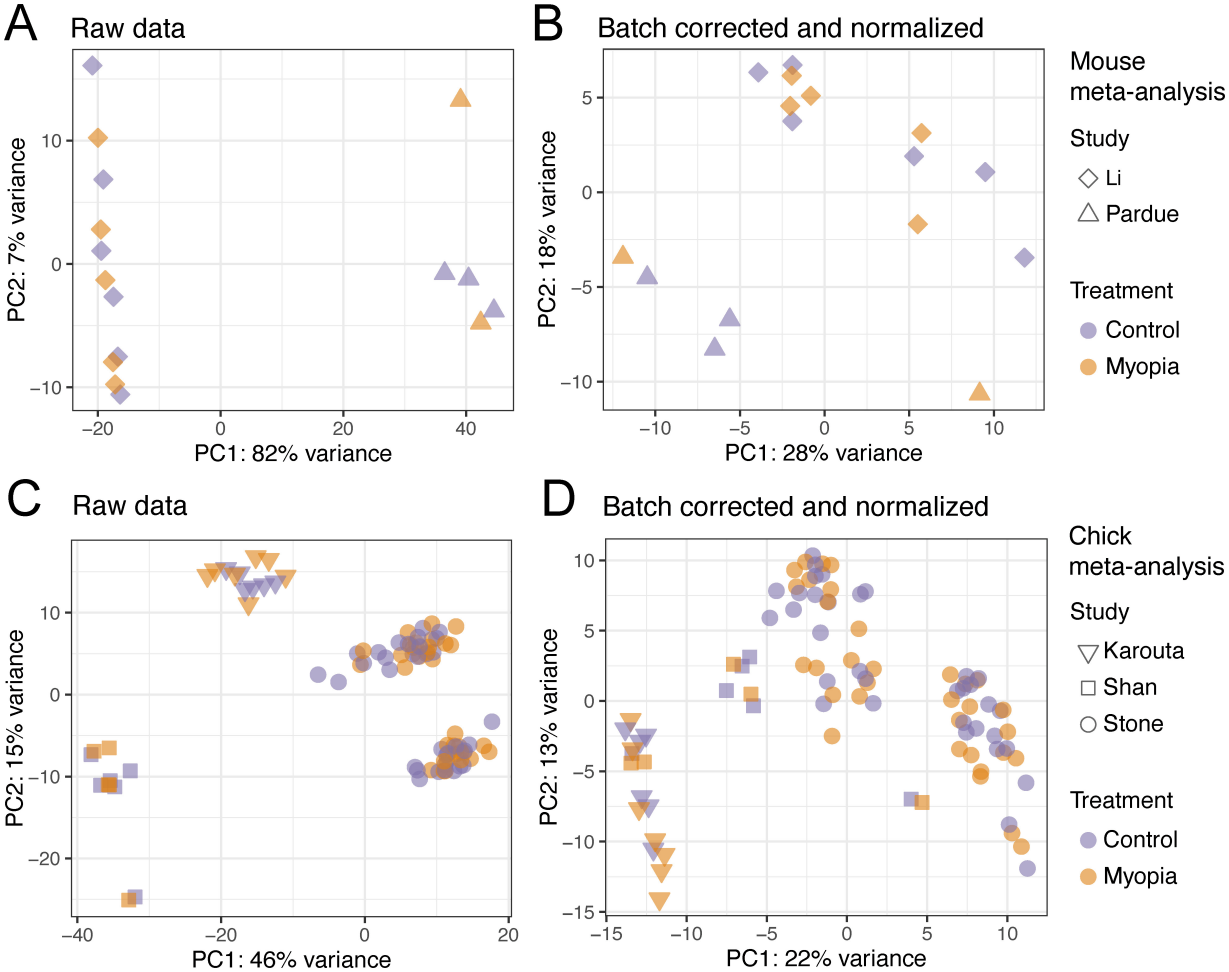
Principal component analysis of the samples included in the RNA-sequencing meta-analysis. The raw RNA-Seq datasets of the studies using the mouse model of myopia **(A)** and chick model of myopia **(C)** before any processing. The RNA-Seq samples of the mouse **(B)** and chick studies **(D)** after batch correction and normalization.

Next, we combined the two studies conducted in mice and the three studies conducted in chicks separately by applying batch correction and normalization using ComBat-seq (30). Before batch correction and normalization, we observed the expected batch effects, revealed by the sample distribution in PCA (Figures 3A, C). After data processing, the samples were clustered significantly closer, while a certain level of segregation between samples remained (Figures 3B, D).

**Figure 4:**
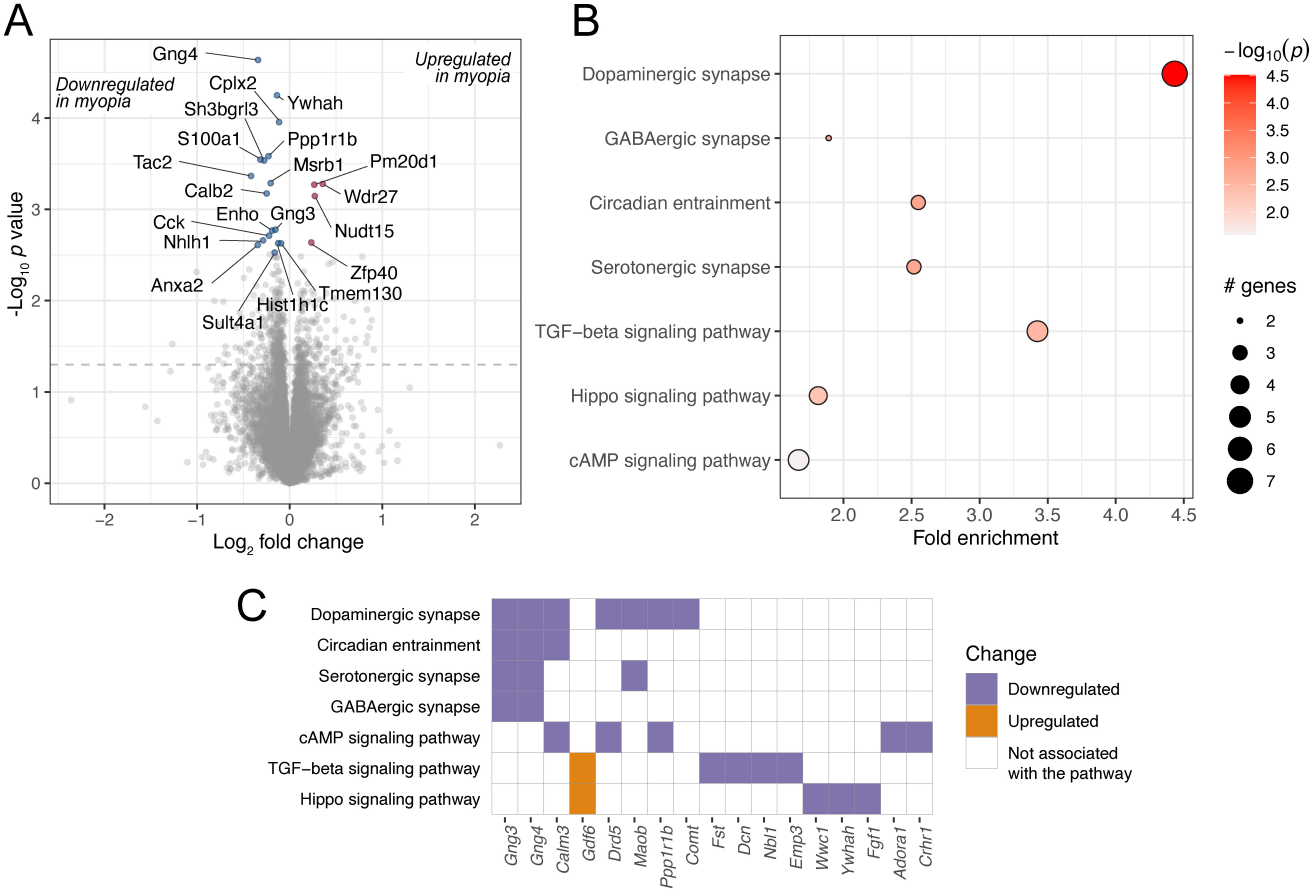
Genes and pathways differentially regulated in mouse retinas in response to experimental myopia. **(A)** A volcano plot of genes differentially regulated in response to experimental myopia in mouse retinas, indicating the log_2_ effect size and unadjusted log-transformed *p* values. The gray dashed horizontal line indicates an unadjusted *p* value of 0.05. A selection of genes significantly changing are highlighted in red (upregulated) and blue (downregulated), with the gene names indicated. **(B)** A selection of KEGG pathways enriched for the differentially regulated genes are highlighted. **(C)** The genes contributing to the pathways illustrated in **(B)** are shown on the horizontal axis, colors represent the directionality of gene expression change in experimental myopia.

The meta-analysis of 16 mouse retinal samples identified 427 differentially regulated genes (unadjusted *p* value < 0.05, Figure 4A, Supplementary Table 6). Among the enriched KEGG pathways, significant changes were observed in signaling pathways such as TGF-beta (Figures 4B, C, Supplementary Table 7), which is critical in ocular growth and myopia development (42, 46). In addition, among the enriched pathways were dopaminergic, GABAergic and serotonergic synapse and circadian entrainment. The genes contributing to these pathways largely overlapped and included G-protein gamma subunit genes *Gng3* and *Gng4* (Figure 4C), which contribute to various receptor signaling pathways (47).

**Figure 5:**
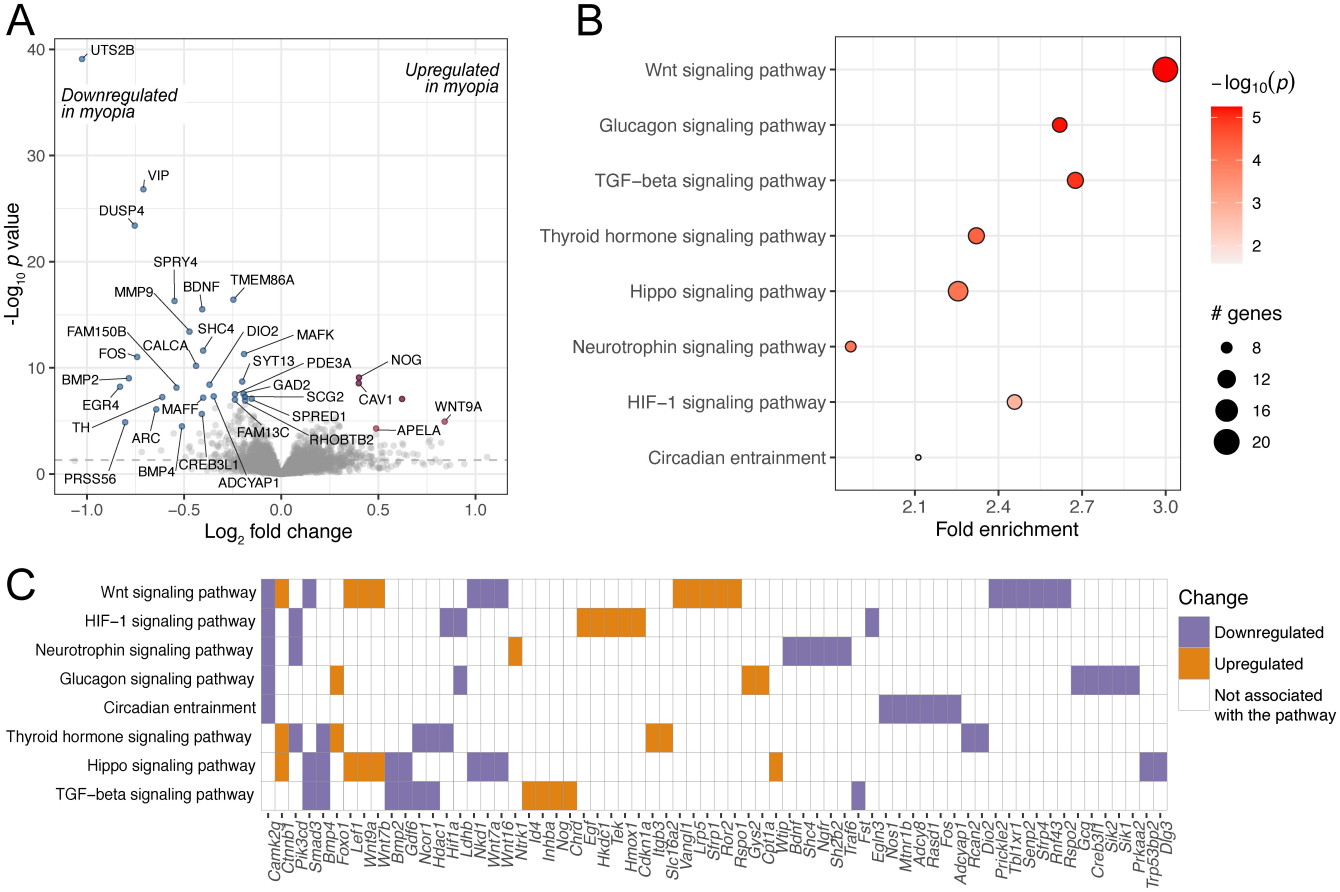
Genes and pathways differentially regulated in chick retinas in response to experimental myopia. **(A)** A volcano plot of genes differentially regulated in response to experimental myopia in chick retinas, indicating the log_2_ effect size and unadjusted log-transformed *p* values. The gray dashed horizontal line indicates an unadjusted *p* value of 0.05. A selection of genes significantly changing are highlighted in red (upregulated) and blue (downregulated), with the gene names indicated. **(B)** A selection of KEGG pathways enriched for the differentially regulated genes are highlighted. **(C)** The genes contributing to the pathways illustrated in **(B)** are shown on the horizontal axis, colors represent the directionality of gene expression change in experimental myopia.

A significantly larger number of samples, a total of 91, were included in the meta-analysis of chick retinal responses to experimental myopia. We found that 1,110 genes were differentially regulated (Figure 5A, Supplementary Table 8), a larger proportion of them (54%), with highest changes in significance, were downregulated. Among those genes was vasoactive intestinal polypeptide (*VIP*), which is expressed in a subset of amacrine cells (48), and its downregulation in response to myopia has been shown in macaque retinas (48), chick retinas (45) and chick retina/RPE complex (49). Furthermore, intravitreal administration of VIP reduced the magnitude of FDM, while VIP antagonists abolished FDM development in chicks (50). The most significantly downregulated gene was urotensin-2B (*UTS2B*; Benjamini-Hochberg adjusted *p* value 1.05E-35). *UTS2B* encodes for urotensin II-related peptide (URP), and the downregulation of prepro-URP has been reported previously in chick retina (45) and retina/RPE complex (49). In addition to its vasoactive properties, URP is also known to induce cell proliferation (51). To our knowledge, the effect of URP in the retina is not well understood. Also among the top downregulated genes was brain-derived neurotrophic factor (*BDNF*), which has been established as neuroprotective agent in the retina (52–54). While the exact role of BDNF in myopia has not been clearly identified to date, lower levels of BDNF have been found in the aqueous humor of myopic individuals (55), and polymorphisms in the noncoding RNA gene BDNF-AS, the antisense RNA of BDNF, have been associated with myopia (56).

In line with a higher number of differentially expressed genes, the gene set was enriched for more pathways (Figures 5B, C, Supplementary Table 9). Similarly to the mouse model, the TGF-beta pathway and circadian entrainment pathways were prominent. Among the pathways that were overrepresented in the genes changing in chick retinas, but not in mouse retinas, were the HIF-1 signaling pathway as well as glucagon signaling.

**Figure 6:**
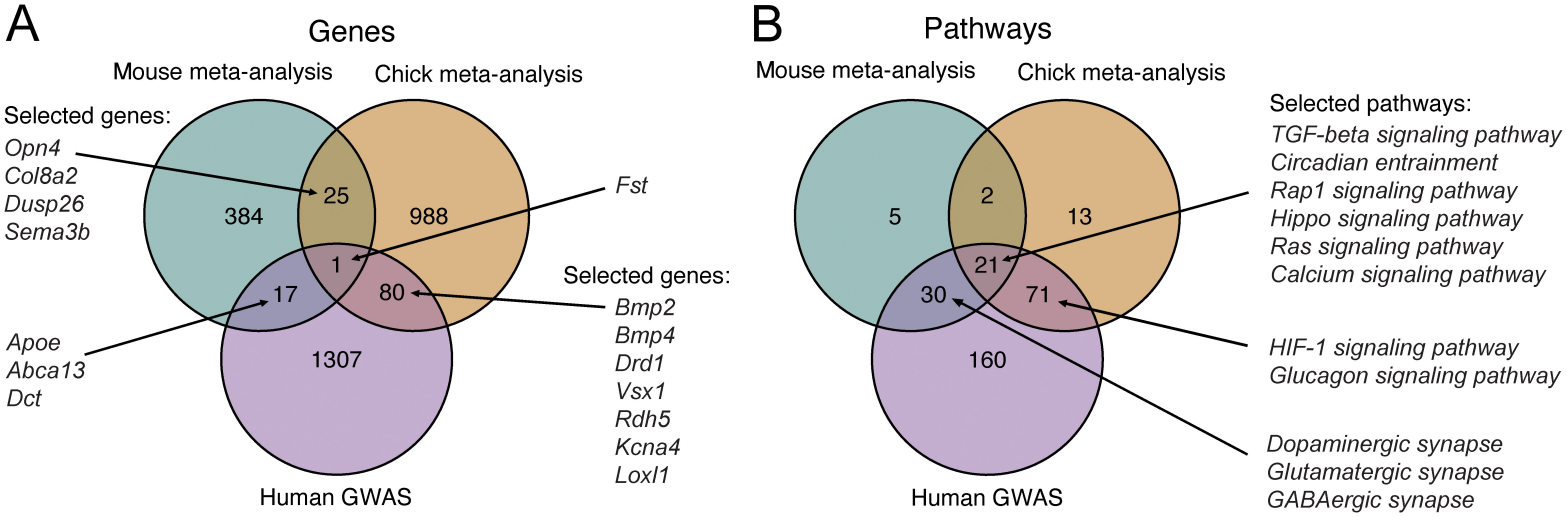
Overlap between genes and enriched pathways differentially regulated in experimental myopia in mouse and chick retinas, and those implicated in refractive error in human GWA studies. **(A)** Venn diagram of genes differentially regulated in experimental myopia in the mouse and chick retina and genes implicated in refractive error in humans show one gene associated with all three gene groups. **(B)** Venn diagram of pathways enriched for genes in **(A)**. A subset of genes and pathways most relevant in the context of the retina and refractive development are illustrated on the graphs. GWAS, genome-wide association study.

Next, we sought to understand to what extent the genes and pathways differentially regulated in the mouse and chick retinas overlap. Furthermore, we wanted to determine how the findings from experimental myopia models compare to the knowledge about refractive error genetics in humans. To this end, we analyzed the results of the meta-analyses in the context of genes implicated in refractive errors in GWAS studies (Supplementary Table 10), defined as being mapped in the proximity of genetic loci associated with refractive error and myopia. Regarding gene-level changes, we found that one gene, follistatin (*Fst*), was downregulated in mouse and chick retinas, and has also been implicated in refractive error in humans (Figure 6A, Supplementary Table 11). The impact of light perception and photoreceptors also seems to be conserved in the mechanisms. In particular, we observed that the gene encoding for melanopsin (*Opn4*) was downregulated in both mouse and chick retinas (Figure 6A). In addition, genes associated with photoreceptor function and phototransduction, such as *Vsx1* and *Rdh5*, were changing in chick retinas and are also implicated in refractive errors in humans (Figure 6A).

We further explored the pathways enriched for the genes changing in mouse and chick retinas, as well as those overrepresented in human refractive error susceptibility genes (Supplementary Table 12), with a subset highlighted for their relevance in retinal and refractive development (Figure 6B, Supplementary Table 13). Pathways associated with all three analyses included, again, the TGF-beta signaling pathway and circadian entrainment processes. These findings reveal the mechanistic similarities between experimental myopia in mouse and chick retinas and our knowledge of refractive error genetics in humans. They highlight the complexities of myopia as a multifactorial condition influenced by both conserved and species-specific pathways.

## 4 Discussion

Myopia is a growing healthcare concern, and a better mechanistic understanding of the disorder is required to design novel and effective management approaches. The increasing number of publicly available studies employing transcriptome profiling in experimental myopia provides the opportunity to combine the studies in meta-analyses, which increases the power of the analysis and allows the detection of consistent changes across experimental protocols and techniques. Here, we performed two meta-analyses on two retinal transcriptome profiling studies of mouse experimental myopia and three retinal transcriptome profiling studies of chick experimental myopia. During the final stages of manuscript preparation, another dataset by Stone et al. (57) was published, which was not included in this analysis.

We identified 427 and 1,110 differentially expressed genes in the mouse and chick retinas upon experimental myopia, respectively. The gene *Opn4*, encoding for melanopsin, a photopigment expressed in the photosensitive retinal ganglion cells, regulating primarily the non-visual light responses (58, 59), was downregulated in the retinas of both species. Melanopsin has been found to have a strong effect on refractive development in the mouse model, where knock-out of the gene results in more hyperopic refractions and an aberrant response to FDM (60, 61). The involvement of visual processing in myopia pathogenesis is indicated by the fact that we identified the gene *Vsx1*, essential for terminal differentiation of subsets of OFF bipolar cells, to be downregulated in chick experimental myopia (Supplementary Table 7) and is also implicated in refractive errors in humans (GWAS Catalog). The knock-out of *Vsx1* has been demonstrated to render mice less susceptible to FDM (62). Another gene differentially regulated in mouse and chick retinas, and involved in visual processing, was dopamine receptor 1 (*Drd1*). It has been shown in mice that the activation of retinal dopamine 1 receptor inhibits FDM development (63).

In the meta-analyses, we found that across species, which also differed in the duration of myopia induction and developmental stage, the TGF-beta pathway was differentially regulated, and the pathway was also enriched for genes associated with human refractive errors. The TGF-beta superfamily comprises cytokines, including TGF-beta, bone morphogenic proteins (BMPs), and several others. We observed downregulation of BMP2 and BMP4 in chick myopia, similar to previous studies (49), and these genes are also implicated in human myopia (Figure 6A). The only gene that was differentially regulated in both mouse and chick retinas and is also implicated in human refractive errors, was follistatin. The primary role of follistatin is to bind and neutralize members of the TGF-beta superfamily, including BMP2 and BMP4 (64), further implying the importance of this pathway in myopia pathogenesis.

Another pathway consistently changing in all three gene sets was circadian entrainment. How exactly circadian rhythms may affect myopia development is unclear, but there is evidence that multiple processes associated with refractive error display daily rhythmicity, including axial length (65, 66) and choroidal thickness (66), and the daily rhythm of axial length is altered in chicks developing experimental myopia (67, 68). In addition, data suggest a difference in behavioral circadian rhythms in myopic individuals. In particular, some studies indicate that myopic children have later sleep timing (69–71), shorter sleep duration (70, 72) and worse sleep quality (70, 73). The mechanisms underlying the association between myopia and circadian rhythms require further investigation.

Among the pathways associated with retinal gene changes in mouse and human myopia was dopamine signaling. While the overall dopamine signaling pathway was not overrepresented in the genes differentially regulated in chick retinas, tyrosine hydroxylase (*TH*), the rate-limiting enzyme in dopamine synthesis, was significantly downregulated in myopic chick retinas (Figure 5A). The relevance of dopamine signaling in myopia pathogenesis has been documented in several studies. For example, systemic administration of dopamine precursor L-DOPA resulted in attenuation of myopic shift in FDM (74), while the knock-out of *Th* in the mouse led to more myopic refractions (75). Furthermore, the dopamine receptor subtypes have been demonstrated to play distinct roles in myopic eye growth (76, 77).

One pathway overrepresented in the genes changing in chick retinas, was the HIF-1 signaling pathway, which has been associated with myopic signals in several studies (78, 79). Another such pathway involved glucagon signaling. The activation of glucagon signaling was demonstrated to inhibit experimental myopia in chicks (80). There is also evidence for the importance of glucagon signaling in the mouse retina. In particular, it has been demonstrated that glucagon increases inhibitory post-synaptic currents in rod bipolar cells in a dopamine-dependent manner, and this effect is abolished in retinas after 3 weeks of FDM (81), suggesting a potential neuromodulatory role for glucagon signaling also in the mouse retina.

In addition to studying myopia using experimentally induced models, other studies have taken advantage of the differences in the extent of myopia between different mouse strains (82), or of mouse models of diseases associated with myopia, such as complete congenital stationary night blindness (cCSNB) (11). Analyzing the retinal transcriptome of different mouse strains that vary in refractive error, Tkatchenko et al. (82) found the involvement of dopamine receptor signaling and phototransduction pathway in baseline myopia. Using three mouse models of cCSNB, Zeitz et al. (11) found that retinal genes differentially regulated were enriched for terms such as mitogen-activated protein kinase (MAPK) pathway and synaptic signaling. Similar to the results we obtained from the meta-analyses, *Bdnf* and *Tgfb2* transcripts were both downregulated in cCSNB models (11). A previous meta-analysis by Riddell et al. (83) studied the transcriptome changes of chick eye tissues in response to optically-induced refractive errors. Interestingly, they found an enrichment of genes associated with the complement cascade (83), which we did not detect. The discrepancy may originate from the tissues included in the analyses, while we only included retinal datasets, Riddell et al. included also the RPE and choroid (83). Collectively, these data highlight both similarities and differences in the molecular pathways underlying myopia across species and models, emphasizing the value of studying diverse experimental systems to gain a comprehensive understanding of myopia development and its underlying mechanisms.

There are several limitations to the meta-analyses presented in this article. First, the number of samples from different sexes was not balanced, in fact, only one study used male and female experimental animals. In that particular study, PCA revealed a strong effect of sex on the retinal transcriptome (Supplementary Figure 1D), and therefore, it is unclear to what extent the results are generalizable across sexes. Second, with this analysis, we identified the most robustly changing retinal transcripts, without differentiating between early and late responses to myopic stimulus, nor the differential effects of FDM and LIM. As new datasets are published and the number of samples increases, these analyses will be very interesting to perform, to further understand the intricacies of the molecular signatures in retinal responses to myopic stimuli.

## Data Availability

The original dataset presented in the study is deposited in the Gene Expression Omnibus repository, accession number GSE284642, and BioProject, accession number PRJNA1200000. These data can be found here: https://www.ncbi.nlm.nih.gov/geo/query/acc.cgi?acc=GSE284642. Publicly available datasets were analyzed in this study. These data can be found in BioProject, accession numbers PRJNA678523, PRJNA832969, PRJNA766764, and PRJNA946718.
