# Supplementary material for: Meta-analysis of retinal transcriptome profiling studies in animal models of myopia
Source: Front Med (Lausanne). 2025 Jan 14;11:1479891. doi: 10.3389/fmed.2024.1479891 (PMC11772478; doi:10.3389/fmed.2024.1479891)
Supplement: Supplementary file 2 [file Image_1.pdf]

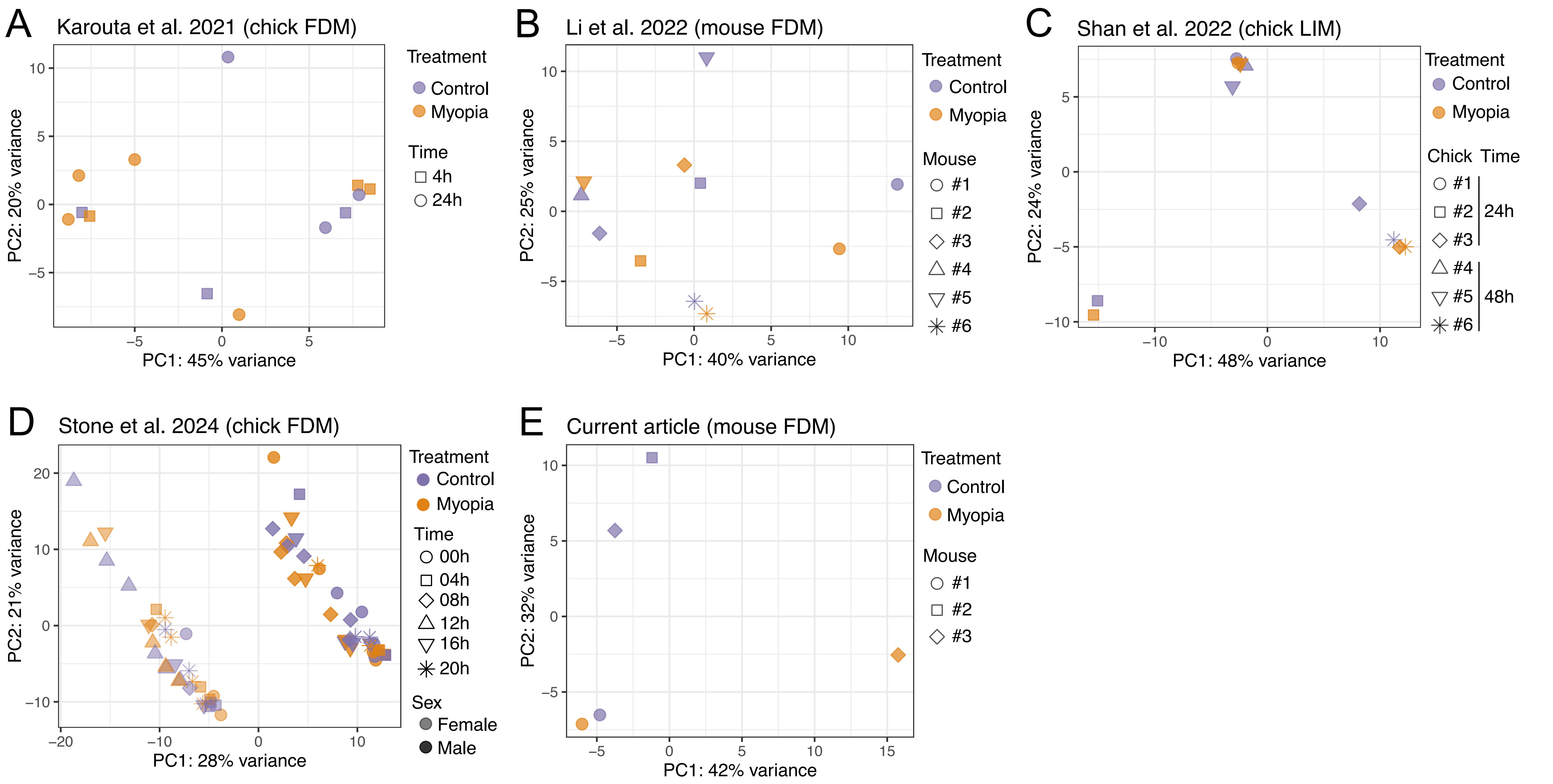

Supplementary Figure 1. Principal component analysis of the RNA-Seq studies included in the meta-analyses.
The principal component analysis considers some of the reported variables included in the experimental design in individual studies included in the meta-analysis. (A) Karouta et al. exposed chicks to form deprivation for 4 and 24 hours and used untreated animals as controls. (B) Li et al. induced myopia in mice with form deprivation using the contralateral eye as a control. (C) Shan et al. induced myopia with a -10 D lens with 24- and 48-hour exposures and used the contralateral eyes as controls. (D) Stone et al. exposed newly hatched chicks to form deprivation for one day and collected thereafter tissue every four hours over one diurnal day. They used the contralateral eyes as controls. Retrospectively, they determined the sex of the chicks. (E) In the RNA-Seq experiment presented earlier in this article, myopia was induced in mice with form deprivation, and the contralateral eyes were used as controls.
FDM – form-deprivation myopia, LIM – lens-induced myopia.
